# Notes and News

**Published:** 1897-11-06

**Authors:** 


### NOTES AND NEWS.

(Collected and Communicated.)

Lady Willox has given £2,000 towards the new Liverpool Consumption Hospital to endow a bed.

The Queen has conferred the Jubilee Medal upon Sir George Duffey, President of the College of Physicians (Ireland) and upon Sir William Thomson, President of the Royal College of Surgeons in Ireland, and upon Mr. Walter Hills, President of the Pharmaceutical Society of Great Britain.

A scheme for the improvement of the infirmary was the form chosen by the inhabitants of Berwick to commemorate the Jubilee. The subscriptions obtained have rendered it possible to entirely remodel the method of heating the building, and the low-pressure system has been introduced throughout. Radiators, with ventilation from without, have been largely adopted.

The completeness and importance of Owens College as a building will be greatly enhanced by the intended additions, to be made through the munificence of the Court of Governors. In addition to the physiological laboratory, it is proposed to erect a large hall wherein to confer degrees and hold college ceremonies. This building will complete the college quadrangle, and much improve its general appearance.

The plans for the new pavilion at the Edinburgh Royal Infirmary have been decided upon. It will be situated at the south end of the infirmary, immediately to the west of the present medical pavilion. The same style of architecture is to be followed as the rest of the buildings. The basement, or *rez de chausée*, will contain a bathing establishment, containing all kinds of baths used in medical treatment. The three floors above will contain wards for female patients, with special wards, operating-room, demonstration and physicians' room, and all necessary offices. The attics will house 14 nurses and nine servants in separate rooms. Altogether the pavilion is to provide accommodation for 97 persons. The total cost of the pavilion is estimated at £40,000.

The foundation-stone for a new union infirmary has been laid at Halifax. Provision for 640 patients and 100 nurses is to be made, and the cost is estimated at £130,000.

A concert in aid of the Charing Cross Hospital will be held on November 19th at the Queen's Hall. Madame Albani has promised to sing, and the Duke of Coburg is interesting himself in the project.

The meeting of Charing Cross Hospital Special Appeal Committee which was to have been held on Wednesday, November 3rd, at the Mansion House, has been postponed to Monday, November 8th, at four o'clock, in consequence of the funeral of H.R.H. the Duchess of Teck having been fixed for November 3rd.

After considerable discussion the Corporation of Dublin has decided to continue the annual grant of £300 to Mercers' Hospital, on the governors of that hospital agreeing to appoint three direct representatives of the Corporation as governors and members of the House Committee.

A strongly signed memorial has been forwarded to the President of the Local Government Board by the Clifton Certified Boarding-Out Committee in favour of continuing the present system of careful inspection of boarded-out children, and of retaining the valuable services of Miss Mason in this department.

The new fever hospital at Darnley, in Scotland, is now completed. It is placed in the centre of the district it is to serve. Accommodation for about 60 patients, including children, is provided. The cost of the hospital is £15,000, which is regarded by those interested as extremely moderate, as it works out at about £357 per bed, whereas in London similar accommodation is quoted as costing over £600 per bed.

The long-discussed and vexed question of a site for the Newcastle Infirmary appears at last to be settled, and the building is to stand on the Leazes, which is the name under which the city recreation ground is known. Mr. John Hall, who is providing the magnificent sum of £100,000 for the building, expressed a preference for the site now chosen, and, when once the infirmary is established, the fear that the pleasures of the people of Newcastle will be curtailed will be probably forgotten, and a worthy pride and a handsome and useful institution will take its place.

The Forster Green Hospital for Consumption, with which is amalgamated the Belfast Hospital for Consumption, is now open. Mr. Green's original intention was to have built an entirely new hospital at a cost of £10,000 could a site be procured. There appears to have been a difficulty about this, and so a large country house and grounds were purchased, and the building altered to meet the requirements of a hospital. The practical difficulties of such a proceeding are obvious, and the alterations and additions when completed amounted to upwards of £8,000. It is always a question whether a dwelling-house can be satisfactorily converted into an institution of the character of a large consumption hospital, and it is not therefore surprising that the expenditure of so large a sum as £8,000 was necessary to secure as good an institution as the Forster Green Hospital now is. The present accommodation is for 40 patients, but the building has been arranged with the intention of making considerable additions in the future.

